# Annexin A7 Levels Increase in Rats With Traumatic Brain Injury and Promote Secondary Brain Injury

**DOI:** 10.3389/fnins.2018.00357

**Published:** 2018-05-29

**Authors:** Fan Gao, Di Li, Qin Rui, Haibo Ni, Huixiang Liu, Feng Jiang, Li Tao, Rong Gao, Baoqi Dang

**Affiliations:** ^1^Department of Rehabilitation, Zhangjiagang Hospital of Traditional Chinese Medicine Affiliated to Nanjing University of Chinese Medicine, Suzhou, China; ^2^Department of Neurosurgery and Translational Medicine Center, The First People's Hospital of Zhangjiagang, Suzhou, China; ^3^Clinical Laboratory, The First People's Hospital of Zhangjiagang, Suzhou, China; ^4^Department of Neurosurgery, The First People's Hospital of Zhangjiagang, Suzhou, China; ^5^Department of Pharmacy, The First People's Hospital of Zhangjiagang, Suzhou, China

**Keywords:** AnnexinA7, traumatic brain injury, secondary brain injury, neuron apoptosis, rat models

## Abstract

The incidence of traumatic brain injury (TBI) has been increasing annually. Annexin A7 is a calcium-dependent phospholipid binding protein. It can promote melting of the cell membrane. Recent studies have shown that it plays an important role in atherosclerosis, other cardiovascular diseases, and a variety of tumors. However, few studies of ANXA7 in TBI have been performed. We here observed how ANXA7 changes after TBI and discuss whether brain injury is associated with the use of ANXA7 antagonist intervention.

**Experimental Results:** 1. After TBI, ANXA7 levels were higher than in the sham group, peaking 24 h after TBI. 2. The use of siA7 was found to reduce the expression of A7 in the injured brain tissue, and also brain edema, BBB damage, cell death, and apoptosis relative to the sham group.

**Conclusion:** ANXA7 promotes the development of secondary brain injury (SBI) after TBI.

## Introduction

The incidence of traumatic brain injury (TBI) has been increasing annually due to an increased number of car accidents and falls by the elderly and has continued to increase year by year (Feigin et al., 2013). The death and disability caused by TBI have also increased accordingly. TBI can cause emotional and cognitive impairment, epilepsy, loss of limb function, and memory impairment, which seriously affects the health of the brain. The development of brain injury after initial injury can significantly aggravate the deterioration and death rate of TBI patients, here called secondary brain injury (SBI) (Hamasaki et al., 2017). At present, little is known about the complex cellular response to the SBI and no effective treatment options are available, especially during its acute phase. The mechanism and time-range study of SBI can provide direction for targeted treatment.

The mechanism of SBI after TBI is very complex. The most important pathological change is tissue edema. Ultrastructural studies have confirmed that the electrolyte and extracellular fluid enter the cerebral nerve cells after injury, resulting in cytotoxic edema. The hemangiogenic edema is caused by the disruption of the blood-brain barrier (BBB). The mechanical damage causes vasoconstriction and the capillary endothelial cells become significantly swollen (Viviani et al., 2014; Wang et al., 2014). Recent studies suggests excitatory neurotoxicity take primary responsibility for SBI.

Annexin is a phospholipid binding protein involved in assembly of the cell skeleton, which forms the basic structural units of the blood-brain barrier (BBB). It has a high affinity with Ca^2+^. It also participates in the formation and opening of Na^+^ and Cl^−^ channels, works on vesicle transportation and the secretion of neurotransmitter (Raynal and Pollard, 1994; Hoque et al., 2014). Annexin A7 (ANXA7, A7) was the first member of the family to be found. It has two subtypes, which molecular weight is 47 and 51 kDa, respectively. When intracellular Ca^2+^ concentration is elevated, A7 protein conformation changes take place. The formation of polymer, with the combination of acidic phospholipid, eventually cause A7 protein to bind to a specific membrane and help some molecules to move through the lipid bilayer (Pollard and Rojas, 1988). There are reports showing that high levels of ANXA7 are expressed in brain tissue after cerebral hemorrhage in rats. This promotes the release of glutamate and N-methyl-D-aspartic acid receptor (NMDA) and mediated excitatory neurotoxicity, eventually promoting SBI.

In conclusion, many studies have demonstrated that ANXA7 promotes brain damage. However, the effect of A7 after TBI is not clear. This study established the rat TBI free fall model, studied in the expression and function of ANXA7 in the TBI secondary brain injury, and the mechanism of action provides a theoretical foundation for the development of new drugs and clinical treatments for TBI patients.

## Materials and methods

### Study design and experimental groups

**Figure 1:**
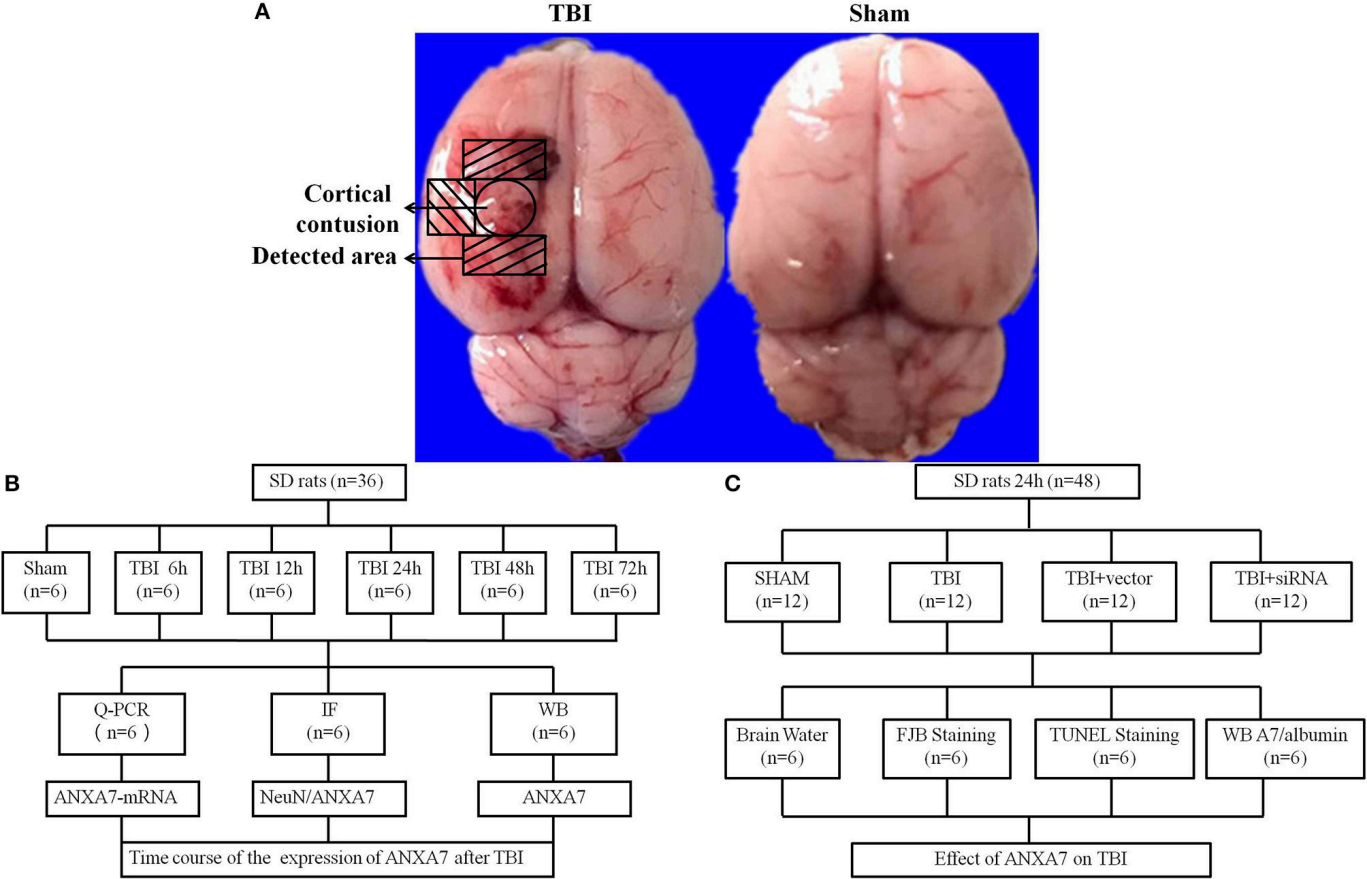
Study design and experimental groups. **(A)** traumatic brain injury rat samples, the representative areas taken for assay; **(B)** experiment group 1 evaluated and located the expression of ANXA7 in TBI rats brain; **(C)** experiment group 2 established the function of ANXA7 in SBI.

Two separate experiments (Figure 1).

Experiment 1: There was no obvious difference in weight, feed intake, and motor ability of all rats. To determine the time course of ANXA7 after TBI, 36 rats (36 surviving out of an initial cohort of 40) were randomly divided into six groups according to a computer-based randomization (EXCEL randbetween function), specifically sham, TBI 6 h, TBI 12 h, TBI 24 h, TBI 48 h, and TBI 72 h (Dash et al., 2016). Brain tissue surrounding the damaged area was sampled. Tissue in the front, close to the frontal lobe was used to perform Western blot analysis (WB). Tissue from the rear part close to the cerebellum was used for double immunofluorescence. Real-time PCR analysis was also performed with tissue from the sides to assess the expression and position of A7 in TBI rat brains (Figure 1B).

Experiment 2: To establish the role of A7 in TBI brain injury, 48 rats (48 surviving out of a group of 57) were randomly divided into four groups according to EXCEL randbetween function, specifically sham, TBI, TBI+ vector, and TBI+ siA7. At 24 h after TBI, which was based on the results of experiment 1, the rats were killed and damaged brain tissue was collected. Neurological testing was examined in all groups before decollation. We randomly selected 6 rats from each group for brain edema evaluation. Other rats were studied using Western blot analysis, terminal deoxynucleotidyl transferase–mediated dUTP nick-end labeling (TUNEL) staining and fluoro-jade B (FJB) staining to measure the expression of A7 and albumin, neuronal apoptosis, and necrosis. Brain tissue from the front area near the damage was used for WB, and tissue from the back was prepared into frozen sections (Figure 1C).

The experiment abides by the blind method strictly. All the samples were encoded by an independent investigator. The experimenters were blinded to all the sample types during the analysis.

### Animals

Here, 97 male Sprague-Dawley rats weighing 280–300 g were purchased from the Animal Center of Soochow University (Suzhou, China), of which 84 were used for statistical analysis. Animals were housed in 12-h light/dark cycles at a controlled temperature and humidity with free access to food and water. All experimental protocols were approved by the Institutional Animal Care and Use Committee of Soochow University and were performed in accordance with guidelines of the National Institutes of Health Guide for the Care and Use of Laboratory Animals.

### Traumatic brain injury model

The TBI model was established using a freefall method (Liu et al., 2016). Rats were intraperitoneally anesthetized with 4% chloral hydrate (400 mg/kg) and fixed using a stereotactic instrument. A 5 mm parietal bone window was made behind the cranial coronal suture next to the midline using a bone drill, keeping the dura intact. A copper cylinder (4 mm in diameter, 5 mm in height) was placed in the bone window. A steel rod weighing 40 g with a flat-end was dropped into the copper cylinder from a height of 25 cm. The rats were allowed to recover until their heart rate and breathing returned to normal after a short pause. We carefully sterilized and stitched the wound. We placed the rats in a warm place and allowed them to recover completely. Sham group rats went through exact same procedure as the TBI group without the 40 g steel rod being dropped through the cylinder (Figure 1A).

### Drug injection

Twenty-four hours before TBI, rats were anesthetized and placed in a stereotaxic apparatus. We made a hole on the left side of the lateral ventricle, located exactly 1.5 mm posterior and 1.0 mm lateral to the bregma (Paul et al., 2015). SiA7 were configuration by 8 μl transfection reagent and 8 μl siA7 (0.52 μg/ul). Sixteen microliters siA7 and 16 μl vehicle (0.26 μg/ul) was injected by microinjection though the hole at a rate of 0.5 μL/min, 4.0 mm beneath the skull. Eventually, the incision was sutured and the rats were allowed to recover.

### Real-time PCR

Total RNA was isolated from brain tissue around the injured area using Trizol reagent (Invitrogen, US) in accordance with the manufacturer's instructions. According to the protocol provided by manufacturer (Thermo, US), complementary DNA (cDNA) was synthesized using 1 μg of the total RNA. Then, real-time PCR was performed using a QuantStudio™ Dx Real-time PCR Instrument (Life Technologies Corporation, US) with a PowerUp™ SYBR™ Green Master Mix Kit (ThermoFisher, US). The phases are briefly described as follows: the template was denatured at 95°C for 2 min, followed by 40 cycles of amplification (95°C for 15 s, 60°C for 15 s, 72°C for 1 min). All samples were analyzed in triplicate. The expression of GAPDH messenger RNA (mRNA) served as an internal reference for each sample, and the relative mRNA expression levels of the target gene were calculated by relative quantification (2^−ΔΔCT^). The primers were as follows: Anxa7, 5′-CCCTGTTCATGCCTCCTACA-3′ and 5′-CACACGCTCTTGAGTTCCTG-3′, GAPDH, 5′-TGGCCTTCCGTGTTCCTACC-3′, 5′-TCTTCCACCACTTCGTCCGC-3′.

### Western blot analysis

Protein extraction from whole-cell lysates of ipsilateral brain were obtained by gently homogenization in RIPA lysis buffer with phosphatase inhibitors (Beyotime, China) with further centrifugation at 13,000 g at 4°C for 20 min. The supernatant was collected and the protein concentration was assessed using the bicinchoninic acid (BCA) method with a Pierce™ BCA Protein Assay Kit (Thermo, US). Equal amounts of extracted proteins were loaded and subjected to electrophoresis on 12% SDS-polyacrylamide gels (Beyotime, China) and then transferred onto polyvinylidene difluoride (PVDF) membranes (Millipore, US). Blocking buffer with 5% defatted milk was used to block the membranes for 1 h at room temperature. These samples were then incubated with following antibodies overnight at 4°C: rabbit anti-A7 (1:1,000, Abcam, US), mouse anti-β-actin (1:10,000, Sigma, US), and chicken anti-albumin (1:1,000, Abcam, US). The membranes were then incubated with horseradish peroxidase-conjugated secondary antibodies for 2 h at 4°C, including goat anti-rabbit IgG-HRP (Invitrogen, US), goat anti-mouse IgG-HRP (Invitrogen, US) and goat anti-chicken IgG-HRP (Invitrogen, US). Immunoblots were finally probed with an Immobilon™ Western Chemiluminescent HRP Substrate (Millipore, US) and visualized with an imaging system (Bio-Rad, US). All data were analyzed using ImageJ software.

### Immunofluorescence staining

The rat brains were removed and fixed in 4% paraformaldehyde at 4°C for 24 h. Samples were dehydrated step by step with 15 and 30% sucrose in phosphate-buffered saline (PBS, pH 7.4) for 24 h, respectively, then embedded in OCT compound (Sakura, US) and frozen at −80°C until use. Frozen coronal slices (15 μm) were sectioned by using freezing microtome and Leica DMi8 (Leica Microsystems, Germany) and mounted on poly-L-lysine-coated glass slides. After three rounds of washing in 1% Triton-PBS buffer to rupture cell membranes, sections were blocked with 10% goat serum for at least 1 h at room temperature, and then incubated at 4°C overnight with primary antibodies: rabbit anti-A7 (1:100, Abcam), mouse anti-Neuron (1:200, Millipore, USA). Secondary antibodies, including Alexa Fluor 488 donkey anti-rabbit IgG antibody (Invitrogen, US) and Alexa Fluor 555 donkey anti-mouse IgG antibody (Invitrogen, US) were incubated for 1 h at room temperature at a dilution of 1:1,000. The sections were observed with a laser confocal microscope Leica DMi8 (Leica Microsystems, Germany) and pictures were taken using LAS X software.

### Brain water content

The brain water content was measured in the second experiment using the wet/dry method (Brockman et al., 2013). After surgery and separation of the brains from surrounding tissues, the brains were divided into ipsilateral and contralateral frontal and quickly weighed to get the wet weight. Then, the samples were placed in a 100°C oven for 24 h to obtain the dry weight. The percentage of brain water content (%) was calculated as [(wet weight – dry weight) / (wet weight)] × 100%.

### Tunel staining

Apoptosis was detected using terminal deoxynucleotidyltransferase-mediated dUTP nick end labeling (TUNEL) staining according to the manufacturer's protocol (Abcam, US). Frozen brain tissue sections were soaked in 4% polyformaldehyde/PBS for 15 min, then shifted into protease K working fluid incubating for 5 min. The samples were immersed in 4% polyformaldehyde/PBS for another 5 min and cleaned with wash buffer twice for 5 min each. We covered the brain slices in DNA labeling solution and stored them in a wet box away from light for 1 h. We washed the slices, added the antibody solution, and placed the samples in a dark, wet box for 30 min. We washed the samples in deionizing solution for 5 min. The samples were allowed to air dry and then sealed with DAPI. We observed the slides with a laser confocal microscope Leica DMi8 (Leica Microsystems, Germany) and took pictures using LASX software.

### Fluoro-jade B staining

Fluoro-Jade B (FJB) staining was conducted as stipulated in the manufacturer's instructions (Millipore, US). After incubation with 1% sodium hydroxide in 80% alcohol for 5 min and 70% alcohol for 2 min, the frozen section were then transferred to a solution of 0.06% potassium permanganate for 10 min. Then, slides were immersed in 0.0004% fluoro-jade dye staining solution (0.1% acetic acid) for 20 min followed by rinsed in deionized water. The sections were washed and dried in an oven at 50°C for 5–8 min. The sections were then cleaned by immersion in xylene for at least 1 min and then coverslipped with Distyrene Plasticiser Xylene (DPX), a non-aqueous non-fluorescent plastic mounting medium. The sections were observed with a laser confocal microscope Leica DMi8 (Leica Microsystems, Germany) and pictures were taken using LAS X software.

### Statistical analyses

All data were analyzed using SPSS 18.0 software. Non-parametric testing was used in the brain water test. When other data were normally distributed, one-way ANOVA was used to compare different groups, *T*-test was used between two groups. The results were expressed as means ± standard deviations. *P* < 0.05 was considered indicative of statistically significant differences; *P* < 0.01 was considered indicative of highly statistically significantly differences.

## Results

### The expression of ANXA7 protein level in brain after TBI

**Figure 2:**
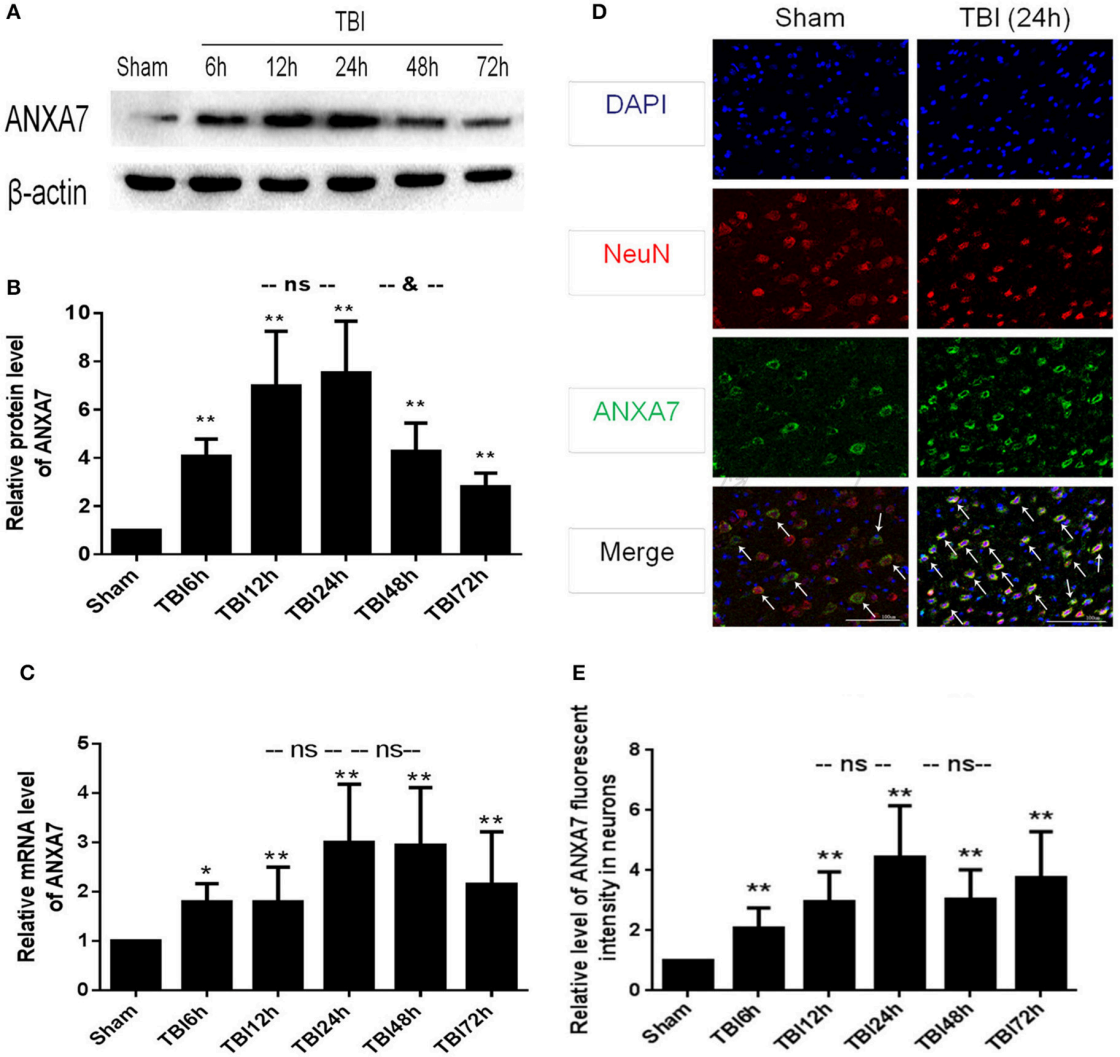
ANXA7 protein level, mRNA level are increased after TBI in rats, and located in neurons. **(A,B)** ANXA7 protein level in brain after TBI. Sample of different time points were analyzed by western blot; β-actin served as a loading control. Protein levels were quantified with Image J software, and mean values for sham group were normalized to 1.0. Date = means ± SD. ^**^P < 0.01 vs. sham; ^&^*P* < 0.05 vs. TBI 24 h; ns *P* > 0.05 vs. TBI 24 h. *n* = 6. **(C)** ANXA7 mRNA level in brain after TBI. Brain tissue collected at different time points were analyzed by Q-PCR. RQ were calculated and mean values for sham group were normalized to 1.0. Date = mean ± SD. ^*^P < 0.05 vs. sham; ^**^P < 0.01 vs. sham; ns *P* > 0.05 vs. TBI 24 h. *n* = 6. **(D,E)** ANXA7 in neurons near the damaged area after TBI. Double immunofluorescence analysis of brain tissue using antibodies against ANXA7 (green) and NeuN (red); nuclei were labeled with DAPI (blue). Quantitative analysis of NeuN and A7 double positive neurons. Date = mean ± SD. ^**^P < 0.01 vs. sham; ns *P* > 0.05 vs. TBI 24 h. Bar = 100 μm. *n* = 6.

Western blot results are shown in Figures 2A,B: Group TBI 6 h, 12 h, 24 h, 48 h, and 72 h had significant differences from the sham group (*P* < 0.01); the TBI 12 h group showed no difference from the 24 h group (*P* > 0.05), the TBI 24 h group was different from the TBI 48 h group (*P* < 0.05). This indicates that undamaged brains have low levels of A7 protein. Six hours after TBI, the expression of A7 was significantly higher than baseline, peaking at 12–24 h. Forty-eight hours after TBI, A7 expression began to decline, and there is still a significant expression after 72 h.

### The expression of ANXA7 in mRNA level in brain after TBI

Q-PCR results are shown in Figure 2C: Groups TBI 6 h, 12 h, 24 h, 48 h, and 72 h all differed from the sham group (*P* < 0.05). Groups TBI 12 h and 48 h group showed no differences from the 24 h group (*P* > 0.05). This indicated that A7RNA was expressed in the sham group at a low level. This level had increased by 6 h after TBI, and significantly increased at 12 h. The high level expression continued to 48 h. There was still significant expression after 72 h.

### The expression of ANXA7 in neurons around the damaged area after TBI

If results are shown in Figures 2D,E: Group TBI 6 h, 12 h, 24 h, 48 h, 72 h has significantly differences with sham group (*P* < 0.01), TBI 12 h, 48 h group has no differences from the 24 h group (*P* > 0.05). This represented A7 expressed in neurons. The sham group showed a low level of expression, with a significant increase 6 h after TBI and a peak at 24 h. The high level of expression was sustained from 12 to 72 h.

### The effect of siA7 intervention on A7 protein expression after TBI

**Figure 3:**
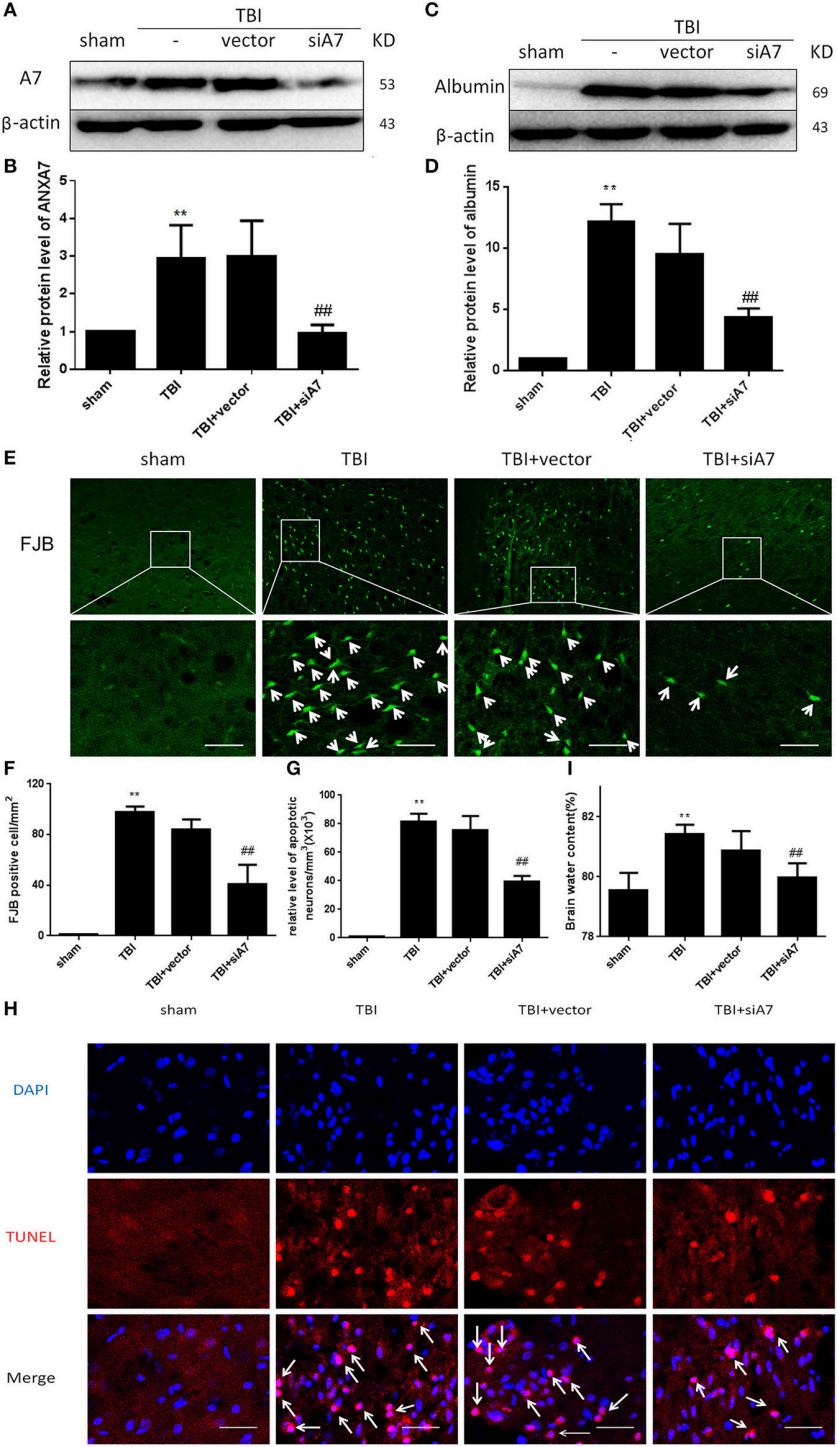
SiA7 reduce ANXA7 in the injured brain tissue and reduce brain injury in rats after TBI. **(A,B)** Confirm the impact of siA7 on ANXA7 expression. A7 was detected in sham, TBI, TBI + vector, and TBI + siA7 groups 24 h after TBI by western blot. Date = means ± SD. ^**^P < 0.01 vs. sham, ^##^*P* < 0.01 vs. TBI. *n* = 6. **(C,D)** The integrity of BBB in TBI rats was improved after siA7 intervention. Albumin was detected by western blot. Date = means ± SD. ^**^P < 0.01 vs. sham, ^##^*P* < 0.01 vs. TBI. *n* = 6. **(E,F)** Neuron death was detected by FJB staining (green). FJB-positive cells/mm^2^ was quantified. Data = mean ± SD. ^**^P < 0.01 vs. sham, ^##^*P* < 0.01 vs. TBI. Bar = 50 μm. *n* = 6. **(G,H)** Neuronal apoptosis was detected with the TUNEL assay. Immunofluorescence analysis was performed with TUNEL (red), nuclei were labeled with DAPI (blue). TUNEL - nuclei positive neurons/mm^3^ was quantified. Date = means ± SD. ^**^P < 0.01 vs. sham; ^##^*P* < 0.01 vs. TBI. Bar = 50 μm. *n* = 6. **(I)** Brain water content was calculated as [(wet weight – dry weight)/(wet weight)] × 100%. Date = means ± SD. ^**^P < 0.01 vs. sham; ^##^*P* < 0.01 vs. TBI. *n* = 6.

Western blot siA7 results are shown in Figures 3A,B: The TBI group showed significant differences from the sham group (*P* < 0.01); TBI showed no differences from the TBI+vector group (*P* > 0.05); the TBI+siA7 group showed significant differences from the TBI group (*P* < 0.01). This indicates that A7 has a low level of protein expression in the sham group that significantly increases after TBI and is significantly reduced after siA7 intervention.

### The integrity of BBB in TBI rats after siA7 intervention

Western blot analysis with albumin are shown in Figures 3C,D: The TBI group showed significant differences from the sham group (*P* < 0.01); TBI has no differences with TBI+ vector group (*P* > 0.05); TBI+siA7 group has significant differences from the TBI group (*P* < 0.01). These results confirmed that the blood brain barrier had been significantly damaged after TBI injury, and the integrity of the BBB was significantly improved after the intervention of siA7.

### Neuron death in TBI rats treated with siA7 injection

FIB staining results are shown in Figures 3E,F: The TBI group showed significant differences from the sham group (*P* < 0.01); TBI showed no differences from the TBI+ vector group (*P* > 0.05); the TBI+ siA7 group showed significant differences from the TBI group (*P* < 0.01). The degree of necrosis of neurons in rats after TBI was significantly greater than in the sham group, and significantly reduced after siA7 intervention.

### Neuronal apoptosis in TBI rats treated with siA7 injection

TUNEL staining results are shown in Figures 3G,H: The TBI group has significantly differences with sham group (*P* < 0.01); TBI showed no differences from the TBI+ vector group (*P* > 0.05); the TBI+ siA7 group showed significant differences from the TBI group (*P* < 0.01). Results showed that the rate of apoptosis in rat neurons increased significantly after TBI and decreased significantly after siA7 intervention.

### Cerebral edema index in TBI rats treated with siA7 injection

Wet/dry method on brain edema are shown in Figure 3I: The TBI group showed significant differences from the sham group (*P* < 0.01); TBI showed no differences from the TBI+ vector group (*P* > 0.05); the TBI+ siA7 group showed significant differences from the TBI group (*P* < 0.01). The results indicated that the cerebral edema in TBI was significantly higher 24 h after TBI, which was significantly different from that of the sham operation group, and the cerebral edema index of the intervention group of the siA7 was significantly lower.

## Discussion

ANXA7 exists in many organs throughout the body, including the brain, heart, liver, parotid gland, spleen, lung, normal reticulocyte, and skeletal muscle (Herr et al., 2003; Guo et al., 2013). Recent studies have shown that ANXA7 plays a very important role in atherosclerosis, other cardiovascular diseases, and a variety of tumors (Tincani et al., 1998; Turnay et al., 2005). Results also showed ANXA7 to be widely expressed in brain tissue. During different periods of embryonic development, ANXA7 subcellular localization in brain cells changed from the cytoplasm to the nucleus (Rick et al., 2005). In the cortex of the adult brain, ANXA7 is located in the cytoplasm but not in the nucleus of vertebral body cells and in the top dendrites (Rick et al., 2005). There is a small amount of expression in the cytoplasm and nuclei of the astrocytes (Zhou et al., 2011). In the hippocampus of adrenal excision mice, ANXA7 was mainly located in glial cells, not in neurons or astrocytes (Moga et al., 2005).

When the concentration of cell calcium ions increases, the subcellular localization of ANXA7 shifts. These ions form a polymer to foster the combination with acidic phospholipids, eventually binding to specific parts of the molecule and moving through the lipid bilayer membrane, mediating the release of neurotransmitters and vesicle transport (Hoque et al., 2014). ANXA7 is expressed so widely and participates in basic physical and pathological activities. However, only a few studies of ANXA7 in brain disease have been reported.

In this experiment, we assessed the expression and function of A7 in TBI, ascertained the distribution of A7 in neurons in rats after TBI, and confirmed that A7 showed the most expression after TBI around the damaged brain tissue at both the mRNA and protein levels. The level of A7 was found to change over time, and the peak time of expression is consistent with clinical practice. By using siANXA7 to reduce the protein level of ANXA7 in brain tissue, the rates of brain edema, BBB damage, neuronal apoptosis, and death all declined. It was confirmed that ANXA7 promotes the development of SBI after TBI.

There are a few limitations to this study. The mechanism underlying the role of ANXA7 in TBI brain injury is unclear. ANXA7 mainly acts according to the Ca^2+^ concentration, and binding to the cell membrane. It can change the permeability of the cell membrane and promote protein release or transfer. In the case of Gerelsaikhan, the Snap-23 stabilized snare-complex plays an important role in neurotransmission (Gerelsaikhan et al., 2012). It is mainly distributed in the postsynaptic membrane in brain, and is the primary material engaged in the transportation of N-methyl-D-aspartic acid receptor (NMDA) receptors (Zhou et al., 2015). NMDA receptor is a subtype of glutamate receptor, which is the most important receptor to mediate Excitatory neurotoxicity (Lai et al., 2014; Newport et al., 2015). It can be speculated that A7 may participate in the process of excitatory neurotoxicity and promote nerve cell apoptosis in the SBI after TBI. The specific mechanism still needs further study. We expect this A7 study can help further understanding the mechanism of TBI, and the suppressive drugs of A7 may be helpful for clinical TBI treatment.

## Conclusion

The results demonstrate that ANXA7 promotes the development of SBI after TBI. Based on this finding, we propose that ANXA7 might be a key physiological active substance after TBI brain damage, also we suggest that it could be a therapeutic target to SBI following TBI.

## Author contributions

BD and RG designed the study. FG, DL, LT, QR, HN, FJ, and HL performed the research, collected and analyzed the data. FG wrote the paper. All authors discussed the results and revised the manuscript.

### Conflict of interest statement

The authors declare that the research was conducted in the absence of any commercial or financial relationships that could be construed as a potential conflict of interest.
